# Key gene screening and diagnostic model establishment for acute type a aortic dissection

**DOI:** 10.3389/fgene.2025.1586880

**Published:** 2025-04-24

**Authors:** Yue Pan, Zhiming Yu, Xiaoyu Qian, Xuesong Zhang, Qun Xue, Weizhang Xiao

**Affiliations:** Department of Cardiovascular Surgery, Affiliated Hospital of Nantong University, Nantong, Jiangsu, China

**Keywords:** bioinformatics, diagnostic model, drug prediction, machine learning, type a aortic dissection

## Abstract

**Background:**

Aortic dissection, particularly acute type A aortic dissection (ATAAD), is a life-threatening cardiovascular emergency with alarmingly high mortality rates globally. Despite advancements in imaging techniques like computed tomography angiography (CTA), delayed diagnosis and incomplete understanding of molecular mechanisms persist, contributing to poor outcomes. Recent studies highlight the role of immune dysregulation, vascular smooth muscle cell (VSMC) apoptosis, and metabolic-epigenetic interactions in AD pathogenesis, underscoring the need for novel biomarkers and therapeutic targets.

**Objective:**

This study aims to identify critical genes and molecular pathways associated with ATAAD, develop a multi-omics diagnostic model, and evaluate potential therapeutic interventions to improve clinical outcomes.

**Methods:**

Transcriptome datasets from the Gene Expression Omnibus (GEO) database were analyzed using differential expression analysis, weighted gene co-expression network analysis (WGCNA), and machine learning algorithms (SVM, Random Forest, LASSO regression). Functional enrichment and immunoinfiltration analyses were performed to explore biological pathways and immune cell interactions. External dataset validation and PCR testing of clinical samples (n = 9) were conducted to confirm gene expression differences. A nomogram diagnostic model was constructed and evaluated for predictive accuracy.

**Results:**

Six core genes were identified: *Ccl2*, *Cdh8*, *Hk2*, *Tph1*, *Npy1r*, and *Slc24a4*, with four (*Ccl2*, *Hk2*, *Tph1*, and *Npy1r*) showing significant differential expression in clinical validation. Functional enrichment revealed associations with immune cell migration, vascular development regulation, extracellular matrix pathways, and the PI3K-Akt signaling pathway. Immunoinfiltration analysis demonstrated increased infiltration of B cell precursors, resting NK cells, and M2 macrophages in ATAAD tissues, negatively correlating with core gene expression. The nomogram model exhibited high diagnostic precision (AUC=0.935, 95% CI: 0.908–0.963), supported by calibration and decision curve analyses.

**Conclusion:**

This study identifies key molecular markers and pathways in ATAAD pathogenesis, emphasizing the role of immune dysregulation and extracellular matrix remodeling. The multi-omics diagnostic model provides a novel tool for early screening, potentially reducing mortality through timely intervention. These findings advance the understanding of aortic dissection mechanisms and offer actionable targets for future research and clinical applications.

## 1 Introduction

Aortic dissection (AD) is characterized by a rupture of the media within the aortic wall due to bleeding, leading to the separation of the wall’s layers and the formation of both a true lumen and a false lumen, with or without communicating branches (Erbel et al., 2014). Specifically, the ascending aorta falls under the classification of Stanford type A (Nienaber and Clough, 2015). Acute type A aortic dissection (ATAAD) represents a life-threatening emergency. Research by Hirst et al. in the 1950s revealed that untreated ATAAD patients faced a mortality rate of 21% within the first 24 h after symptom onset, rising to approximately 37% within 48 h (Harris et al., 2022). While prompt surgical intervention markedly enhances survival rates in ATAAD cases, the surgical mortality remains considerable (Daily et al., 1970). Even in experienced cardiac centers, the surgical mortality rate for ATAAD ranges between 10% and 35% (Nienaber and Clough, 2015). A recent retrospective study conducted by Eremia, I.A. et al. Showed that for patients with type A aortic dissection, the 1-year survival rate of the surgical group was 87.5%, while the 1-year survival rate of the conservative management cohort was 30% (Eremia et al., 2025). Consequently, early identification and timely intervention are crucial in managing aortic dissection. The most common symptom of ATAAD is the abrupt onset of severe chest or back pain. Chest pain in more common in patients with type A dissection (79%) compared to type B dissection (63%), whereas back pain is more frequent in type B dissection patients (43% vs 64%, respectively) (Hagan et al., 2000). Although the primary symptoms have remained largely unchanged, diagnostic methods are continually evolving (Zhu et al., 2020). For patients with suspected aortic dissection, imaging diagnosis is the primary approach, as it can swiftly confirm or exclude the diagnosis, classify the extent of the dissection, and assess the urgency of the situation (Ince and Nienaber, 2007). Currently, blood tests play a secondary role in the evaluation of patients suspected of having aortic dissection (Gawinecka et al., 2017). Toru Suzuki et al. found that D-dimer, commonly used to diagnose pulmonary embolism, can also aid in the diagnosis of aortic dissection. The critical threshold of 500 ng/mL, typically employed to exclude pulmonary embolism, is similarly applicable for ruling out aortic dissection within the first 24 h of symptoms onset (Suzuki et al., 2009). Through proteomics analysis, Zhao et al. found that the diagnostic model of type B aortic dissection based on five different proteins (IL-6, GDF-15, CD58, LY9 and SIGLEC-7) as biomarkers has good performance (Zhao et al., 2025). Pu et al. found that PANoptosis-related genes (*Gadd45b*, *Cdkn1a*, and *Sod2*) in vascular smooth muscle play a crucial role in aortic dissection (Pu et al., 2025). However, there is still a lack of research on biomarkers point at type A aortic dissection that can be used for auxiliary diagnosis.

This study aimed to investigate the changes in gene expression associated with the pathophysiology of ATAAD and to develop potential new diagnostic biomarkers. We meticulously analyzed two gene expression datasets from the Gene Expression Omnibus (GEO) and identified differentially expressed genes (DEGs) from ascending aorta samples. Basic modules related to ATAAD were established, and DEGs were further refined using support vector machine recursive feature elimination (SVM-RFE), random forest (RF), and least absolute shrinkage and selection operator (LASSO) algorithms. Subsequently, we developed and validated a predictive model for clinical ATAAD diagnosis based on hub genes. The diagnostic utility of the four hub genes and the nomogram model was thoroughly validated using receiver operating characteristic (ROC) curves. The identification of these four central genes and the accompanying morphological maps significantly enhances the diagnosis of ATAAD in high-risk patients, thereby contributing to a deeper understanding of the etiology of ATAAD.

## 2 Materials and methods

### 2.1 Data acquisition and processing

**FIGURE 1:**
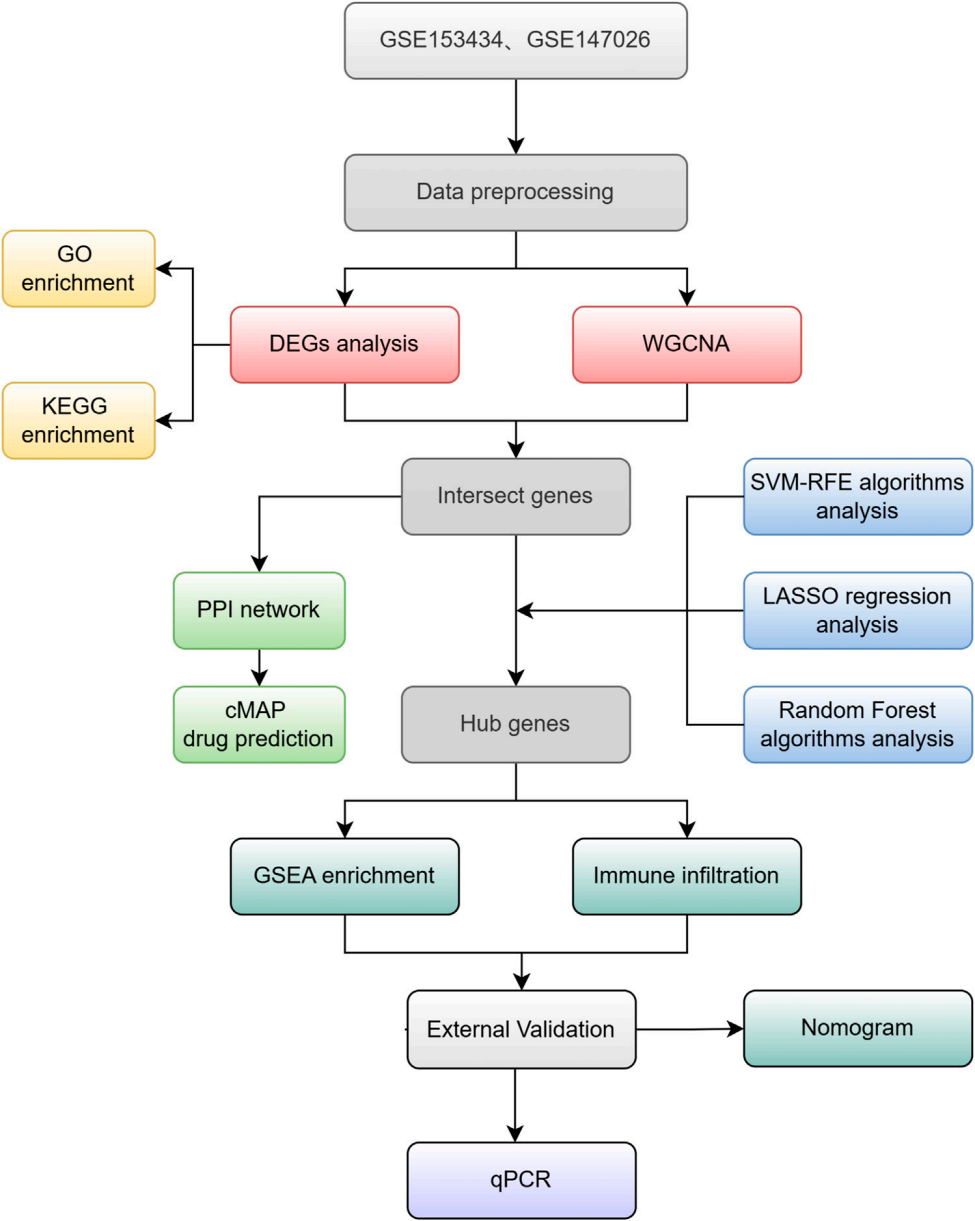
Study Workflow.

The gene expression datasets GSE153434 (Zhou et al., 2020), GSE147026 (Zhou et al., 2021), and GSE52093 (Pan et al., 2014), pertaining to type A aortic dissection, were obtained from the GEO database (https://www.ncbi.nlm.nih.gov/geo/). Specifically, GSE153434 comprises 10 normal samples and 10 samples from patient with ATAAD, while GSE147026 includes 4 normal samples and 4 ATAAD samples. These datasets exhibit robust quality control, featuring complete matrices and comprehensive clinical information. The detection data are publicly available and accessible, and include matrix information that can be normalized effectively. Standard gene expression normalization and log2 conversion were applied to the RNA-seq data. Subsequently, the limma and combat packages in R software were employed for further data normalization. For validation purposes, GSE52093 was utilized, containing 5 normal control samples and 7 ATAAD samples. The research flow chart is presented in Figure 1.

### 2.2 Differential expression analysis

The limma software package in R software was utilized to analyze the differential expression between ATAAD samples and normal control samples (Chen et al., 2017). The truncation standard was adjusted to *p* < 0.05, | log fold change (FC) | >2. The volcano plots and heatmaps are generated using the ggplots and pheatmap software packages, respectively.

### 2.3 Enrichment analysis

To determine the biological relevance of genes and functions, functional enrichment analysis of DEGs was performed. Gene ontology (GO) is a database for annotating gene functions, including molecular functions, biological pathways, and cellular components. Additionally, the Kyoto Encyclopedia of Genes and Genomes (KEGG) pathway database is employed to analyze gene function and related advanced genomic functional information. To gain deeper insights into the role of hub genes, GO and KEGG analysis were performed using the clusterProfiler and DOSE software packages (Ritchie et al., 2015). A p value threshold of less than 0.05 is set as the cut-off standard, and the results were presented in the form of bubble diagram and heat diagram.

### 2.4 Analysis of weighted gene co-expression networks

We batch-processed and integrated the datasets from GSE153434 and GSE147026. The evaluation of trait-associated modules was conducted using the WGCNA software package (Weighted Gene Co-expression Network Analysis). Based on the expression profiles, we constructed a topological overlap matrix, applying a soft threshold power of 5 and establishing a minimum module size of 50 to refine the core modules. To guide the module merging process, a height threshold of 0.5 was applied. Subsequently, a Pearson correlation test was performed on the modules, with a significance threshold set at *p* < 0.05. Finally, DEGs were intersected with the genes from the WGCNA hub module to identify potential candidate genes.

### 2.5 Analysis of protein-protein interaction networks

The analysis of DEGs regarding protein-protein interactions (PPIs) was performed using the STRING database (https://cn.string-db.org/). This resource facilitates the identification of connections among target proteins, including direct binding interactions and the overlapping pathways regulating upstream and downstream activities, thereby enabling the construction of intricate PPI networks characterized by complex regulatory dynamics (Liu et al., 2018). Interactions with a comprehensive score exceeding 0.4 were considered statistically relevant. Cytoscape (http://www.cytoscape.org) served as the tool for visualizing the PPI network.

### 2.6 Prediction of drug candidates

The Connectivity Map (https://clue.io) serves as an online resource for assessing the correlations between gene expression profiles indicative of disease and those modulated by various compounds. This tool facilitates a deeper understanding of drug mechanisms and aids in the discovery of novel therapeutic agents. Consequently, the DEGs were submitted to the CMap database to predict small molecule drugs that might offer potential treatment options for ATAAD.

### 2.7 Machine learnings

Machine learning methods, specifically LASSO regression (Vasquez et al., 2016), random forest (Paul et al., 2018) and SVM algorithm (Noble, 2006), can be employed to identify key genes of ATAAD. Following an initially filtering of differentially expressed genes, the glmnet package, randomForest package and e1071 package were utilized to screen out overlapping critical genes.

### 2.8 Analysis of immune cell infiltration

To investigate the variety of immune cells in ATAAD tissues, single sample gene set enrichment analysis (ssGSEA) (Chen et al., 2022) and IOBR package were used for the immune infiltration analysis. This analysis allowed us to compare the infiltration patterns of 22 immune cell types between normal and ATAAD samples. The immune cell types we identified encompassed B cell progenitor cells, B cell memory cells, plasma cells, T cell CD8^+^, T cell CD4^+^ progenitor cells, T cell CD4^+^ resting memory cells, T cell CD4^+^ activated memory cells, T cell follicular helper cells, T cell regulatory (Treg) cells, T cell γδ, NK cell resting cells, NK cell activated cells, monocytes, macrophages M0, macrophages M1, macrophages M2, dendritic cell resting cells, dendritic cell activated cells. Mast cell resting cells, mast cell activated cells, eosinophils, and neutrophils.

### 2.9 The correlation between key genes and infiltrating immune cells

To explore the association between the identified key genes and the infiltrating immune cells, the gene expression data set of ATAAD was analyzed by Spearman correlation analysis using corrplot R package.

### 2.10 Confirmation of essential gene expression in the validation dataset

The mRNA levels of the identified crucial genes were confirmed within the GSE52093 validation dataset. *p* < 0.05 was regarded as statistically significant.

### 2.11 Construction of diagnostic model

The predictive ability of the nomogram model was assessed using a calibration curve. Additionally, decision curve analysis (DCA) was used to evaluate the practical applicability of the model. For the ROC curve, we utilized the pROC software package, and the area under the curve (AUC) was calculated to gauge the diagnostic accuracy of both the hub gene and the nomogram model.

### 2.12 scRNA-seq analysis

Through single cell sequencing analysis, the distribution of four key genes in each cell cluster was explored, and the difference in expression between the disease group and the control group was verified.

### 2.13 Specimen collection and real-time fluorescence quantitative PCR

The criteria for selecting aortic dissection cases were as follows: individuals must be older than 18 years and diagnosed as aortic dissection by aortic CTA angiography, subsequently undergoing aortic artificial vascular replacement. Normal aortic tissue was obtained from patients undergoing coronary artery bypass grafting. The study was conducted according to the Helsinki Declaration. All research protocols and experiments were approved by the Ethics Committee of the Affiliated Hospital of Nantong University, and all participants signed the informed consent.

Total RNA was extracted from aortic wall tissue using Trizol reagent (ACCURATE BIOTECHNOLOGY Company), and mRNA was reverse transcribed using Vazyme's HiScript II Q RTsuperpMix for qPCR (+ gDNA wiper) kit (R223-01). The ChamQ SYBR qPCR premix kit (Q311-02) was used to perform RT-qPCR with β-Actin as the internal reference gene. The detailed primer sequence of this study is shown in Table 1. The relative expression of the gene was calculated using the 2- △△Ct method (Livak and Schmittgen, 2001). *p* < 0.05 was considered statistically significant.

**TABLE 1:** Detailed Primer sequences of this study.

*Hk2*	Forward Primer (5'→3′)	TGC​CAC​CAG​ACT​AAA​CTA​GAC​G
	Reverse Primer (5'→3′)	CCC​GTG​CCC​ACA​ATG​AGA​C
*Tph1*	Forward Primer (5'→3′)	ACG​TCG​AAA​GTA​TTT​TGC​GGA
	Reverse Primer (5'→3′)	ACG​GTT​CCC​CAG​GTC​TTA​ATC
*Ccl2*	Forward Primer (5'→3′)	CAG​CCA​GAT​GCA​ATC​AAT​GCC
	Reverse Primer (5'→3′)	TGG​AAT​CCT​GAA​CCC​ACT​TCT
*Cdh8*	Forward Primer (5'→3′)	AGC​GGA​AAT​GCT​CTT​GGA​TCT
	Reverse Primer (5'→3′)	GCG​GTT​CAA​AAT​TCG​CTG​TTC​T

### 2.14 Statistical analysis

The data were analyzed utilizing R software (version 4.4.3,https://www.r-project.org). Statistically significant differences between groups were determined using Wilcoxon test. Differences were considered statistically significant at *p* < 0.05.

## 3 Results

### 3.1 Differentially expressed gene identification

**FIGURE 2:**
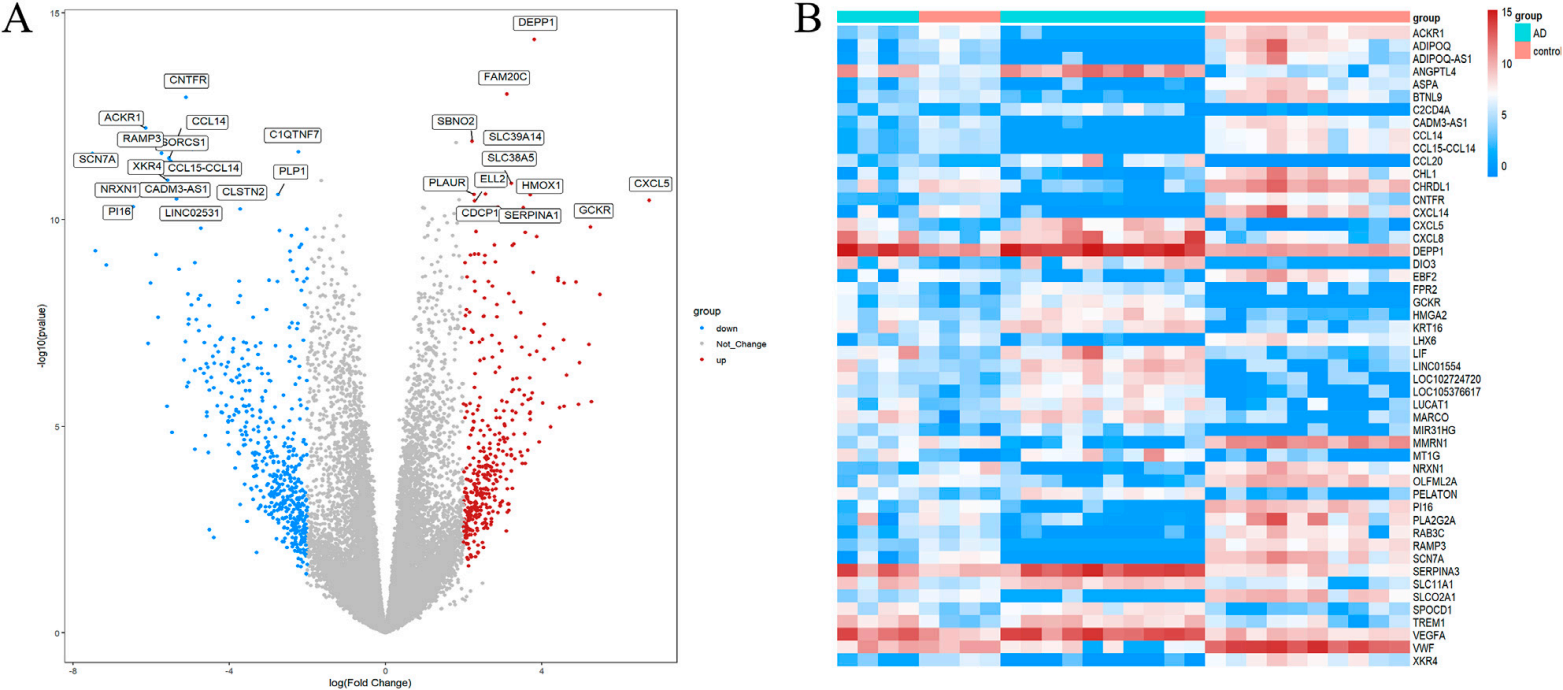
DEG screening between ATAAD and healthy control. **(A)** Volcano graphic visualizing DEGs of ATAAD and normal samples. **(B)**Heatmap of DEGs among normal and ATAAD samples.

Firstly, we analyzed differential expression using two microarray datasets: GSE153434 and GSE147026. Supplementary Figure S1 displays the expression matrix both before and after normalization. We identified a total of 676 DEGs in the combined expression matrix, as illustrated in Figure 2.

### 3.2 Functional analysis

**FIGURE 3:**
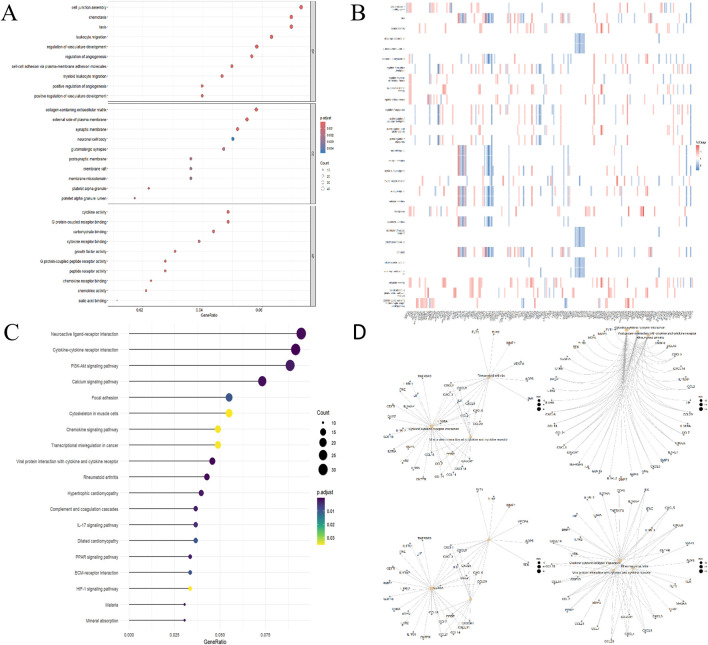
Functional DEG enrichment. **(A)** GO Analytics Bubble Chart (Top 5 in each category). **(B)** Heatmap of the relationship between GO and differentially expressed genes. **(C)** KEGG analysis lollipop plot. **(D)** Network diagram of the relationship between KEGG pathway and differentially expressed genes.

GO analysis revealed 306 biological processes (BP), 33 cellular components (CC) and 44 molecular functions (MF), as shown in Supplementary Table 1. Figure 3A highlights the top 10 GO terms for each category. Notably, the DEGs are significantly enriched in processes such as cell junction assembly, chemotaxis, motility, leukocyte migration, collagen-rich extracellular matrix, regulation of vascular development, regulation of angiogenesis, regulation of membrane potential, and axonogenesis. A heatmap illustrating the correlation between DEGs and the top 50 GO terms is presented in Figure 3B. According to KEGG analysis, DEGs were enriched in pathways such as neuroactive ligand-receptor interaction, cytokine-cytokine receptor interaction, the PI3K-Akt signaling pathway, calcium signaling pathway, focal adhesion, cytoskeleton in muscle cells, chemokine signaling pathway, transcriptional misregulation in cancer, interaction between viral proteins and cytokines and cytokine receptors, and rheumatoid arthritis, as shown in Figure 3C. In addition, Figure 3D displays the association network of the top five KEGG pathways with the DEGs.

### 3.3 Overlap between aortic dissection-related module genes and differentially expressed genes

**FIGURE 4:**
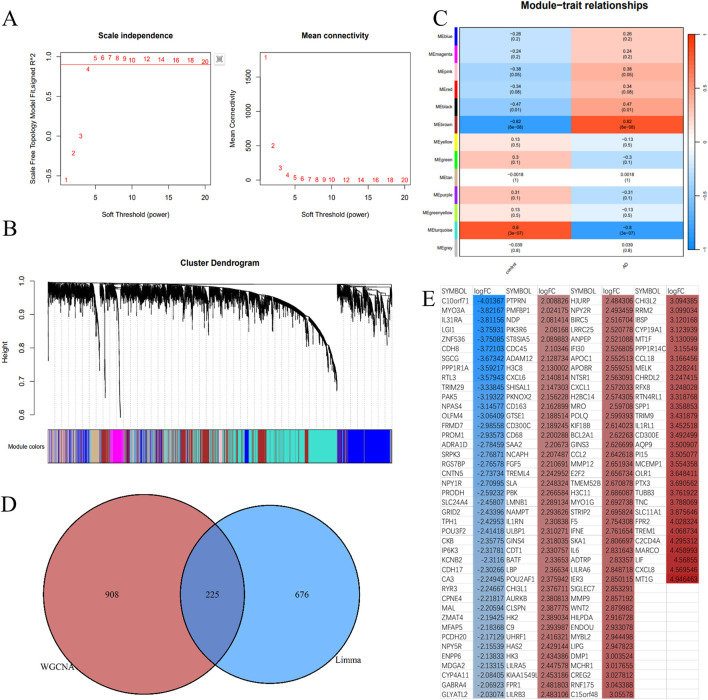
Identification of critical modules by WGCNA. **(A)** Scale-free fit index and mean connectivity for different soft-thresholding powers. **(B)** Topological overlap dissimilarity aggregation of DEGs clusters. **(C)** Module-feature correlations Each row represents a module list, whereas each column represents a clinical characteristic. The first line of each cell includes the associated correlation, while the second line gives the P-value. **(D)** Venn diagram for overlapped genes. **(E)** The overlapping genes were sorted by logFC, with red representing upregulated genes and blue representing downregulated genes in aortic dissection.

A scale-free network with a soft threshold of 5 (*R* ^2^ = 0.91) is constructed and shown in Figure 4A. Following this, we determined the module characteristic genes, calculated the aggregate gene expression level of each module, and grouped them according to their correlation. 13 modules were identified, as illustrated in Figure 4B. Among these, 3 modules were found to be associated with ATAAD: brown (correlation coefficient = 0.82, *p* < 0.0001), black (correlation coefficient = 0.47, P = 0.01), and turquoise (correlation coefficient = −0.8, *p* < 0.0001). For further investigation, a total of 908 ATAAD-related genes identified in the three modules were retained, as shown in Figure 4C. Finally, 225 genes were identified to overlap, as presented in Figures 4D, E.

### 3.4 Identification of the interconnection network between protein diseases

**FIGURE 5:**
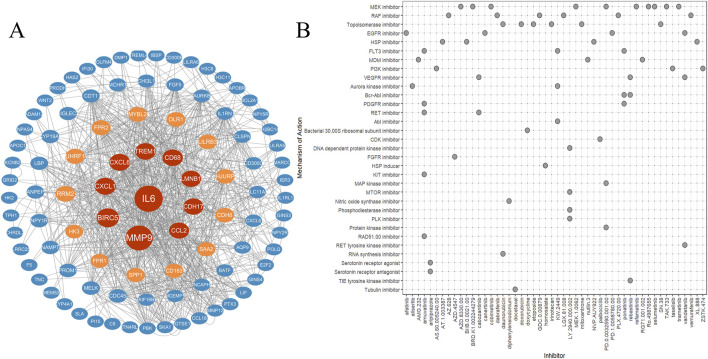
PPI and cMAP. **(A)** Protein-protein interaction network diagram (analyzed online through the STRING website). **(B)** Predict potential small molecule drugs through cMAP and create a heatmap.

The PPI network was developed using STRING database to identify gene cluster modules that surpassed a comprehensive score threshold of 0.4. This network was then visualized with Cytoscape. The hub genes were ranked according to the Betweenness algorithm through the cytoCNA plug-in and subsequently visualized using Cytoscape, as illustrated in Figure 5A. This network comprises 96 nodes and 823 edges, with further details available in Supplementary Table S2.

### 3.5 Prediction of drugs for the treatment of type a aortic dissection

The differentially expressed genes in ATAAD were analyzed through the CMap database to predict potential small molecule compounds that could offer therapeuticy benefits for ATAAD. Compounds exhibiting a negative correlation may hold the potential to alleviate the symptoms associated with ATAAD. The top 50 small molecule drugs (all connectivity scores exceeding 0.7) are presented in Figure 5B.

### 3.6 Identification of key genes

**FIGURE 6:**
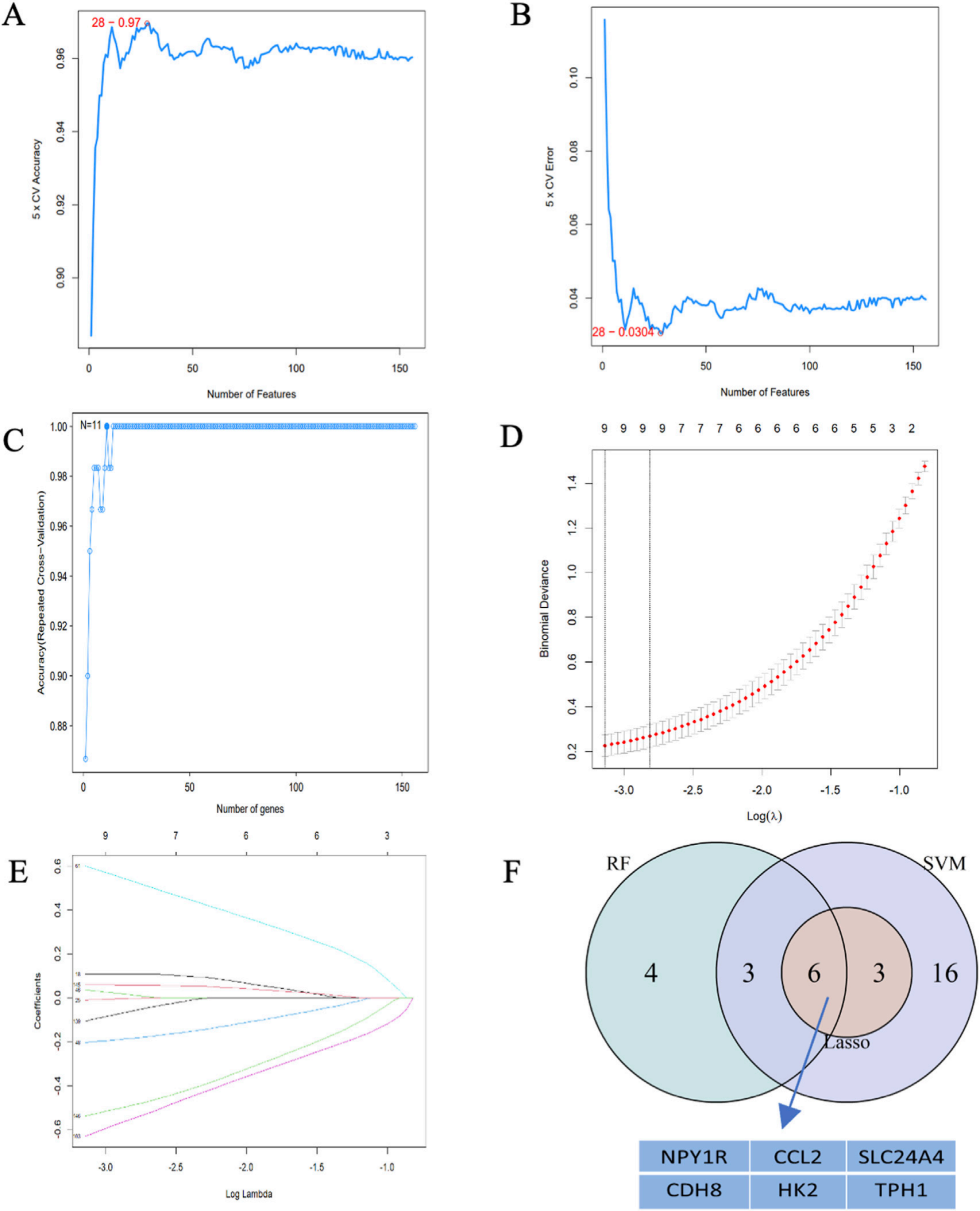
Hub gene identification. **(A)** 28 gene signatures were identified by SVM-RFE analysis with an accuracy of 0.97. **(B)** Error of 0.0304. **(C)** Prediction accuracy of the RF model. **(D)** Cross-validation to select the optimal tuning parameter log(Lambda) in LASSO regression analysis. **(E)** LASSO coefficient profiles of candidate genes. **(F)** Venn diagram of four hub genes shared by the SVM-RFE, RF, and LASSO algorithms.

To discover the characteristics of genes, 225 candidate genes were analyzed by SVM-RFE, RF, and LASSO methodologies. Utilizing SVM, we pinpointed 28 genes with an impressive accuracy of 0.97, as illustrated in Figures 6A, B. Through the application of random forest approach, 11 characteristic genes were filtered, as depicted in Figure 6C. Furthermore, the LASSO regression method revealed 9 gene characteristics, detailed in Figures 6D, E. By examining the overlap among the results obtained from these three techniques, we determined six hub genes: *Ccl2*, *Cdh8*, *Hk2*, *Npy1r*, *Tph1*, and *Slc24a4*, which were represented in Figure 6F. When compared with the control group, the levels of *Ccl2*, *Cdh8*, *Hk2*, and *Slc24a4* were significantly lower in ATAAD samples, whereas *Npy1r* and *Tph1* exhibited notable increases, as shown in Figure 9A. The correlation analysis indicated a strong relationship among these six genes, as illustrated in Figure 9B.

### 3.7 Analysis of the relationship between hub genes and immune cell infiltration

**FIGURE 7:**
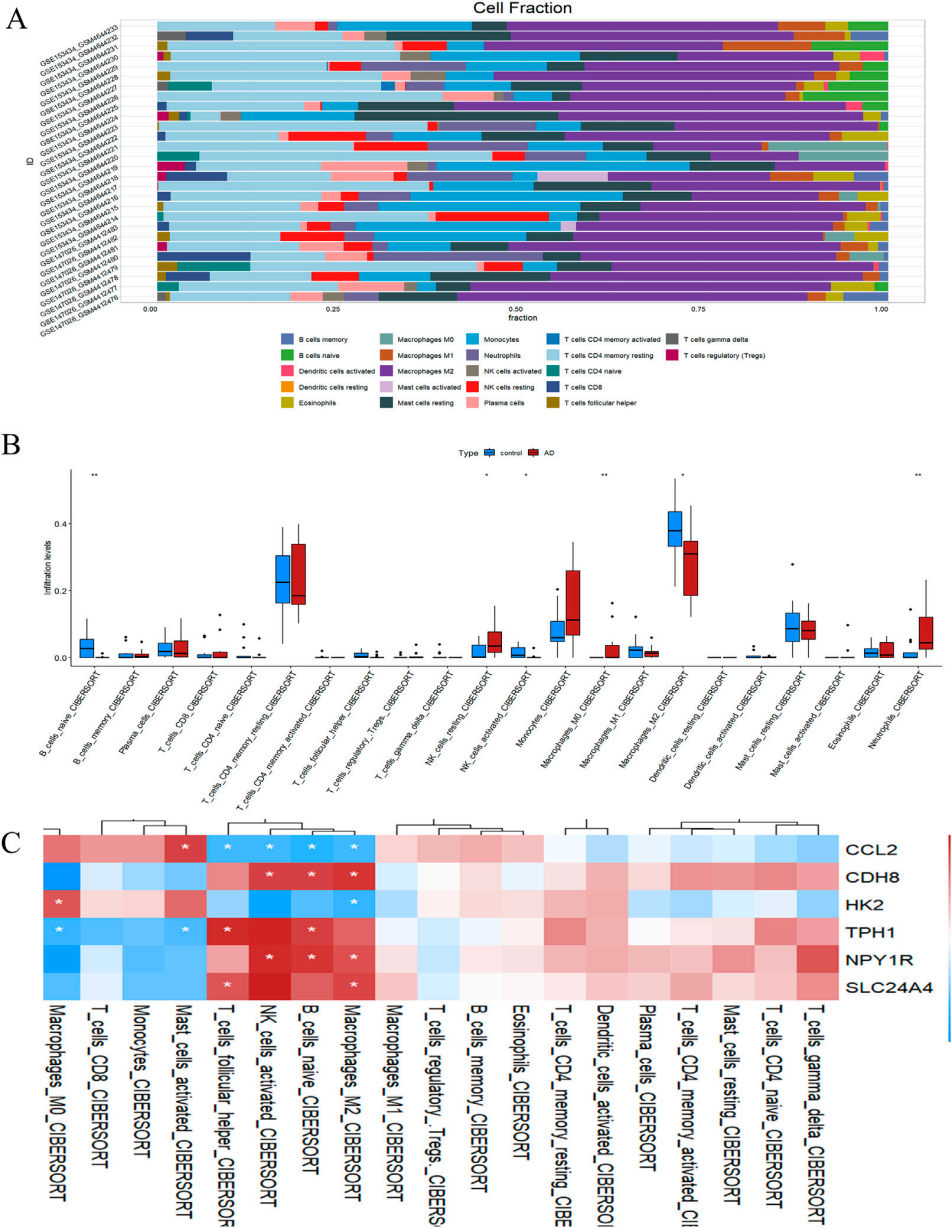
Immune cell distribution in ATAAD. **(A)** Stacking diagram of immune cells. **(B)** Differences in infiltrated immune. **(C)** Correlation analysis between hub genes and immune cells.

To investigate the infiltration pattern of immune cells, we utilized IOBR2, with the immune cell distribution in each sample illustrated in Figure 7A. In ATAAD samples, we observed a significant higher prevalence of B cell precursors, resting NK cells, activated NK cells, M0 macrophages, and M2 macrophages compared to normal samples, as illustrated in Figure 7B. Furthermore, we computed the relationship between the expression levels of hub genes and the infiltration of immune cells. The findings indicated that a substantial majority of the immune cells exhibited a significant negative correlation with hub gene expression, as demonstrated in Figure 7C. These results imply that inflammatory factors might play a crucial role in the initiation and progression of ATAAD, while hub genes could potentially serve as novel regulators in immune responses.

### 3.8 Gene set enrichment of hub genes

**FIGURE 8:**
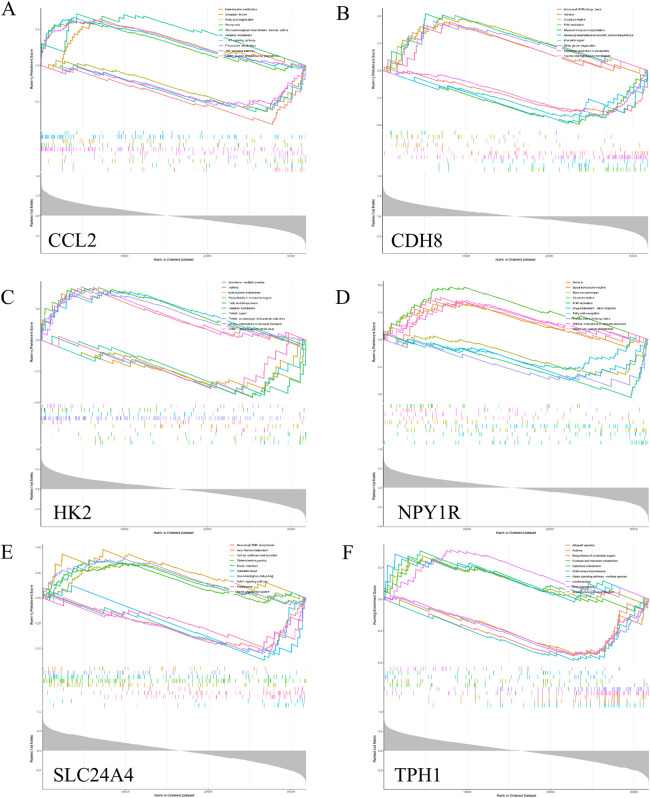
GSEA analysis of hub genes. Top 5 GSEA enrichment in the high and low expression. **(A)** *Ccl2*. **(B)** *Cdh8*. **(C)** *Hk2*. **(D)** *Npy1r*. **(E)** *Slc24a4*. **(F)** *Tph1*.

To further reveal the potential role of *Ccl2*, *Cdh8*, *Hk2*, *Npy1r*, *Tph1* and *Slc24a4*, we performed a single gene GSEA analysis, as shown in Figure 8.

### 3.9 External data set validation

**FIGURE 9:**
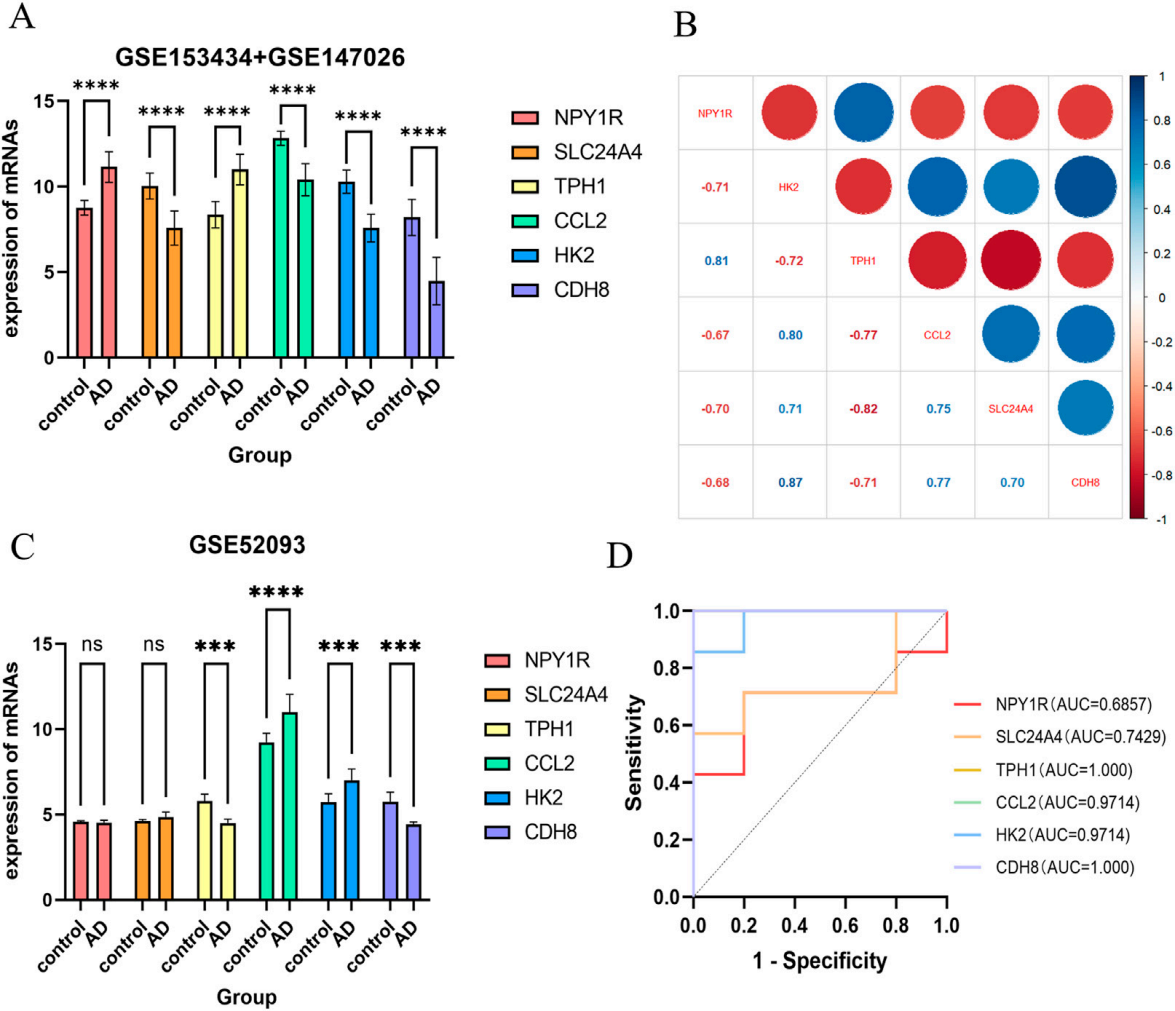
Expression analysis of hub genes. **(A)** Internal validation: expression of six hub genes in ATAAD and control groups. **(B)** Correlation between hub genes. **(C)** External validation (GSE52093): expression of six hub genes in ATAAD and control groups. **(D)** Diagnostic evaluation of hub genes. ROC curve to evaluate prediction accuracy in GSE52093. **p < 0.01, and ***p < 0.001.

Furthermore, we downloaded the data set GSE52093 from the GEO database for external validation of the six hub genes we had identified. Our analysis revealed significant differences in the expression levels of *Ccl2*, *Cdh8*, *Hk2*, and *Tph1* between ATAAD patients and healthy individuals, as shown in Figure 9C. In addition, the ROC curves of six hub genes in this dataset are shown in Figure 9D.

### 3.10 Development and assessment of diagnostic model

**FIGURE 10:**
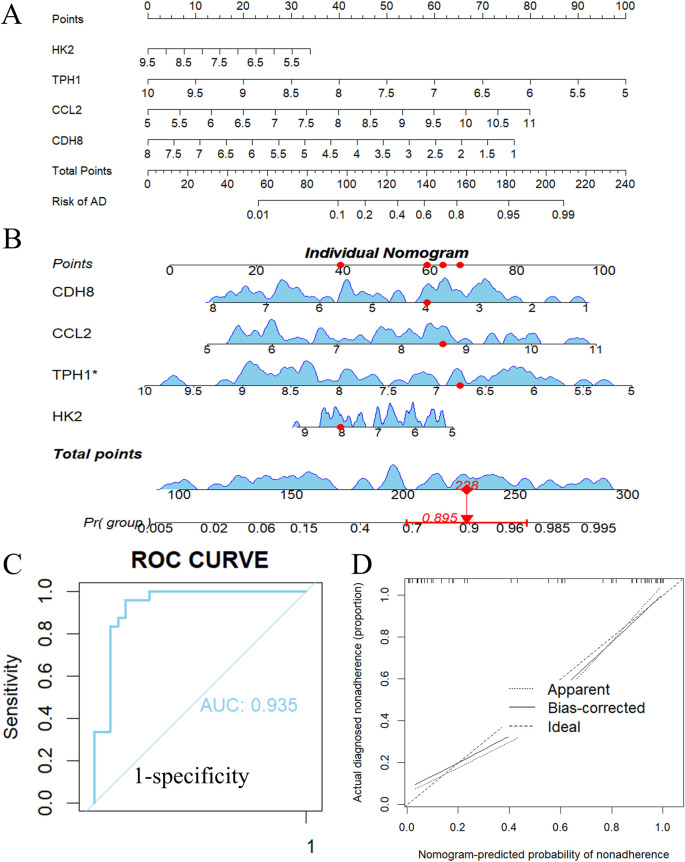
Nomogram model construction for ATAAD. **(A)** Nomogram to predict ATAAD risk. **(B)** Individual nomogram of an ATAAD patient. **(C)** ROC curve to evaluate prediction accuracy. **(D)** Calibration curve evaluation for the diagnostic potential of the nomogram model.

To enhance the sample size, we integrated four data sets: GSE153434, GSE147026, GSE52093, and GSE98770 (Kimura et al., 2017), resulting in a comprehensive dataset of 51 normalized samples. A diagnostic nomogram was created utilizing *Ccl2*, *Cdh8*, *Hk2*, and *Tph1*, as illustrated in Figure 10A. As an example, we applied the nomogram to the aortic dissection case GSM4412480 from GSE147026, generating a nomogram curve for this particular case. The predicted risk of dissection was found to be 89.5%, as depicted in Figure 10B. The area under the curve (AUC) of the nomogram achieved an impressive value of 0.935, with a 95% confidence interval (CI) ranging from [0.908 to 0.963], as presented in Figure 10C. The calibration curve indicates minimal variance between the observed and predicted risks, demonstrating that the nomogram model effectively predicts ATAAD, as shown in Figure 10D. Notably, our diagnostic models exhibited a high degree of accuracy in risk prediction for ATAAD.

### 3.11 Single cell sequencing analysis

**FIGURE 11:**
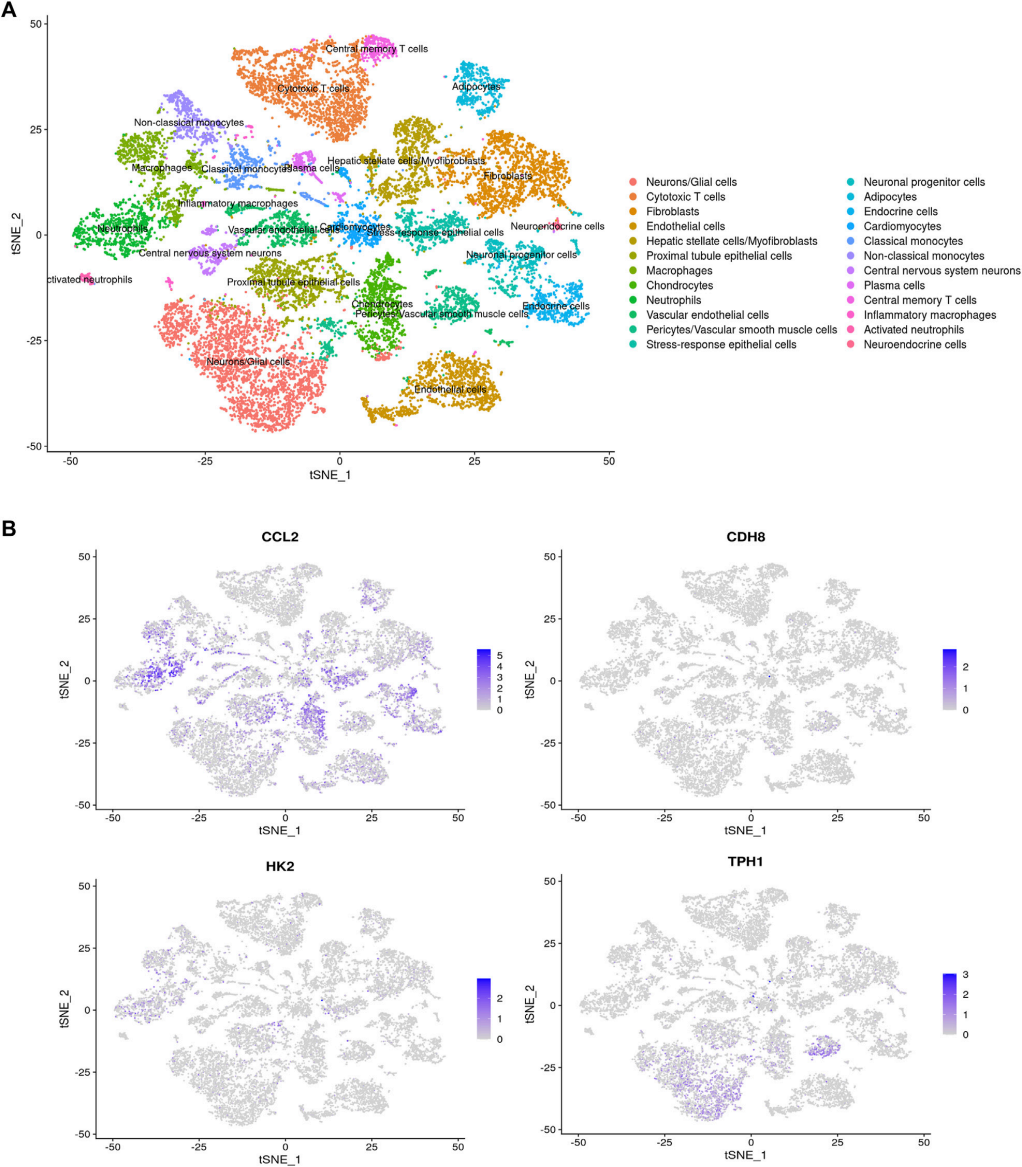
tSNE diagram. **(A)** tSNE distribution map. **(B)** The distribution of four hub genes in various types of cells.

**FIGURE 12:**
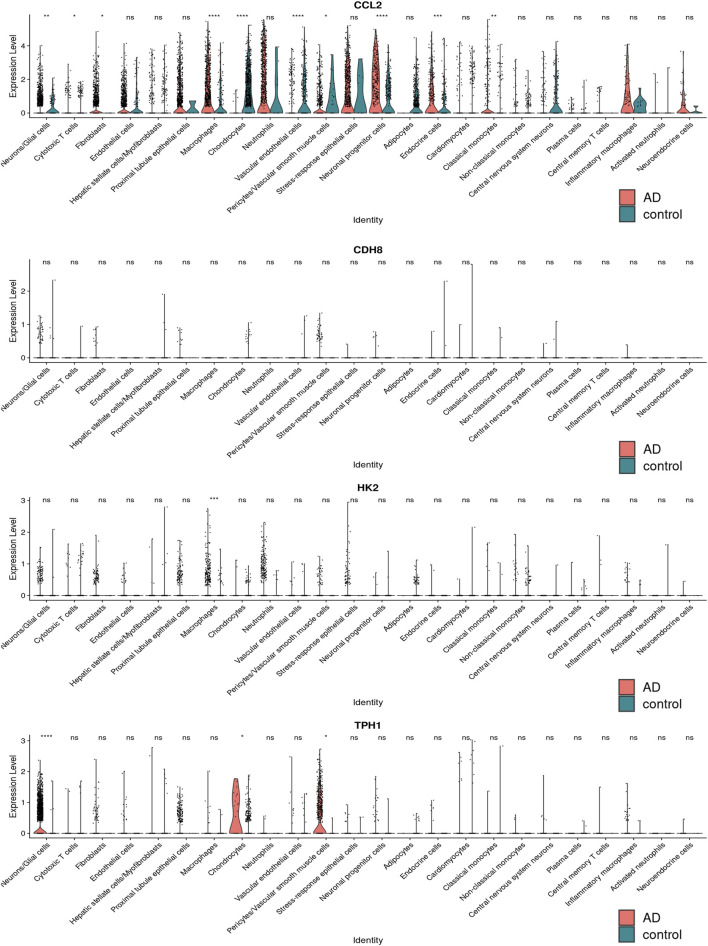
The distribution difference map of four key genes in various types of cells.

The single-cell sequencing data GSE213740 (Zhang et al., 2023)was downloaded from the GEO database, and quality control (screening criteria 200 < RNA-nfeatures <5,000, mt< 5%), batch merging were performed on the data set in R. Then, we carried out dimensionality reduction and cluster analysis on the sorted data, and drew the tSNE diagram, as shown in Figure 11A. The distribution of four key genes in each cell cluster is shown in Figure11B. The expression differences of the four key genes in the AD group and the control group are shown in Figure 12. It can be seen that *Ccl2* is the most widely distributed, and there is a significant difference in expression between the AD group and the control group. In the AD group, the expression of *Ccl2* in macrophages was higher than that in the control group, and the expression in chondrocytes and vascular endothelial cells was lower than that in the control group. Similarly, the expression of *Hk2* in AD was also significantly higher than that in the control group. This suggests that the formation and development of AD is closely related to the inflammatory response. The expression of *Tph1* in neurons/glial cells was the most different, and the expression of *Cdh8* was not significantly different.

### 3.12 Specimen collection and real-time fluorescence quantitative PCR

**FIGURE 13:**
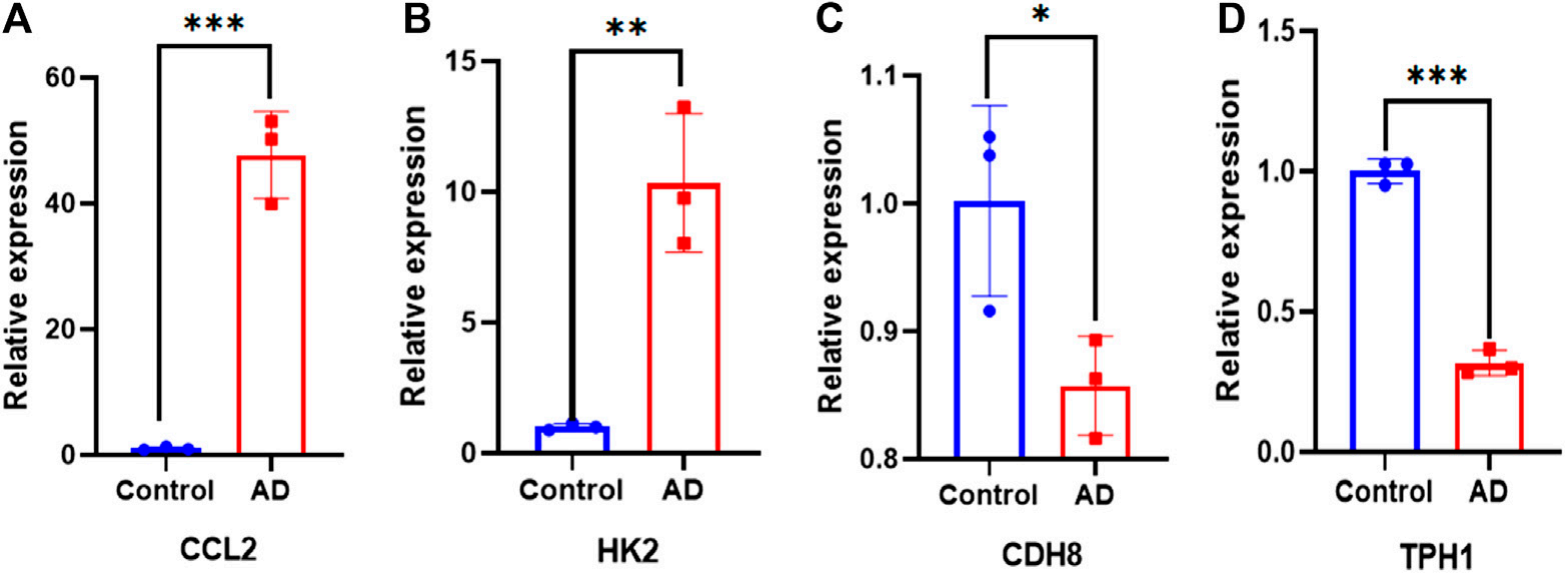
qPCR validation. Comparison of gene expression between ATAAD rat model and control in **(A)** *Ccl2*. **(B)** *Hk2*. **(C)** *Cdh8*. **(D)** *Tph1*. ∗*p* < 0.05, ∗∗*p* < 0.01, and ∗∗∗*p* < 0.001.

A total of 9 clinical samples were included in this study: 6 samples from AD patients and 3 samples from patients undergoing aortic coronary artery bypass grafting as normal control group to further verify the expression of differential genes. As illustrated in Figure 13, there were significant differences in the expression levels of *Ccl2*, *Hk2*, *Cdh8* and *Tph1* between the healthy controls and patients with AD.

## 4 Discussion

ATAAD is a critical disease characterized by high mortality rates, and the prognosis of affected patients is significantly influenced by time-dependent factors. Therefore, timely and accurate diagnosis is crucial for determining patient outcomes (Kimura et al., 2017; Zhang et al., 2023; Rylski et al., 2023; Matthews et al., 2021). This study highlights the development of four hub genes that hold promise as innovative diagnostic tools for ATAAD. Initially, we screened DEGs with significant expression differences and relatively high expression levels in both ATAAD patients and healthy controls. Subsequently, WGCNA was employed to analyze three gene modules that exhibited strong correlations with ATAAD, narrowing down the list from 676 DEGs to 225. We then applied three machine learning methods-support vector machine (SVM), random forest (RF), and LASSO regression-to further identify six hub genes whose expression levels are closely associated with ATAAD: *Ccl2*, *Cdh8*, *Hk2*, *Npy1r*, *Slc24a4*, and *Tph1*. These machine learning techniques facilitated the selection of genes that most significantly contribute to the predictive accuracy of our models.

The inclusion of each gene was further validated through statistical significance testing and prognostic correlation analysis, enhancing the reliability of the multi-gene signature and its biological relevance to the phenotypic outcomes of disease. Finally, our study employed various methodologies to confirm the feasibility of these six hub genes in diagnosing ATAAD. Specifically, we utilized an external gene expression dataset (GSE52093 from the GEO database) and real-time fluorescence quantitative PCR for detection. The diagnostic potential of four hub genes-*Ccl2*, *Cdh8*, *Hk2*, and *Tph1*-was supported by the GSE52093 dataset, which demonstrated their correlation across diverse patient cohorts. In contrast, *Npy1r* and *Slc24a4* exhibited no significant differences in expression. Real-time fluorescence quantitative PCR provided quantitative validation for these findings. The upregulation of *Ccl2* and *Hk2*, coupled with the downregulation of *Tph1* and *Cdh8* in ATAAD patients, further supported our predictions. The differential expression of these hub genes underscores their potential significance for enhancing clinical diagnostic accuracy.

By integrating cross-validation of external datasets with real-time fluorescence quantitative PCR, this study not only confirmed the reliability of the key genes identified through bioinformatics analysis and machine learning methods but also established the clinical applicability of these four hub genes. By introducing these genes, we initially proposed the feasibility of a non-invasive diagnosis for ATAAD and laid the groundwork for further molecular research on this critical condition.

In recent years, RNA sequencing (RNA-seq) has emerged as an indispensable tool for investigating developmental processes and exploring molecular disorders underlying various diseases (Thind et al., 2021; Liu et al., 2021). Furthermore, the integration of bioinformatics analysis and machine learning methods has facilitated the exploration of key genes, potential mechanisms, and therapeutic targets (Nguyen et al., 2022). Numerous studies have focused on the identification of ATAAD-related key genes. For instance, Zhang et al. identified six gene features associated with RNA modification (*Ythdc1*, *Wtap*, *Cfi*, *Adarb1*, *Adarb2*, *Tet3*) that may be utilized for the diagnosis and risk stratification of ATAAD (Zhang et al., 2024). Li et al. identified eight immune-related genes (*Cxcr4*, *Lyn*, *Ccl19*, *Ccl3l3*, *Sell*, *F11r*, *Dpp4*, and *Vav3*) as hub genes, which represent potential biomarkers and therapeutic targets associated with the immune response in ATAAD patients (Li et al., 2022). Similarly, Huang et al. identified seven hub genes related to immune infiltration: *Abcg2, Fam20c, Ell2, Mthfd2, Ankrd6, Glrx* and *Cdcp1,* noting that the expression of four of these genes (*Abcg2, Fam20c, Mthfd2,* and *Cdcp1*) is linked to cardiovascular dysfunction (Huang et al., 2024). He et al. found that BASP1 monocyte subsets play a catalytic role in the formation of AAD and provide a new potential target for its early intervention (He et al., 2025). In contrast to these studies, our research employed a more extensive array of screening methods, resulting in the identification of hub genes with increased reliability. Furthermore, we constructed a diagnostic model that can be initially utilized for the clinical diagnosis of ATAAD. This model also predicts potential targeted small molecule compounds, which may help prevent the onset of ATAAD and alleviate its symptoms. Additionally, the functional elucidation of key genes *Ccl2*, *Hk2*, *Tph1*, and *Cdh8* provides valuable insights into the biological landscape of ATAAD and underscores their potential involvement in the disease’s pathogenesis.

Understanding the effects of key genes in inflammatory CCL2, also known as MCP-1, is crucial given its status as one of the earliest discovered chemokines (He et al., 2025; Matsushima et al., 1989; Yoshimura et al., 1989). CCL2 is a potent pro-inflammatory cytokine that binds to its cognate receptor, CCR2 (Charo et al., 1994). It exhibits robust chemotactic activity towards CCR2^+^ monocytes, macrophages, and CD4^+^ T cells (Carr et al., 1994). Beyond its chemotactic functions, CCL2 also exerts a range of immunomodulatory effects, including systemic inflammation, angiogenesis, and organ fibrosis (Gschwandtner et al., 2019). Previous studies have demonstrated that CCL2 expression is elevated in infarcted myocardial tissue (Chen and Frangogiannis, 2021), promoting cardiac regeneration through the activation of STAT3 signaling, thereby highlighting its potential therapeutic role in myocardial infarction (MI) and related heart failure (Wang et al., 2024). Moreover, CCL2 is significant in cardiovascular disease, which increases its applicability in the context of aortic dissection.

Hexokinase (Hk) serves as a rate-limiting enzyme in aerobic glycolysis, catalyzing the conversion of glucose into the metabolic intermediate glucose-6-phosphate (G-6-P) (Bian et al., 2022). In mammals, four isoforms of the HK family have been identified: HK1, HK2, HK3 and HK4 (Guo et al., 2023; He et al., 2023). HK1 is ubiquitously expressed in all adult tissues, whereas HK2 is primarily expressed in adult muscle and heart tissues, as well as in embryonic-derived cells (Xu et al., 2018). Studies have provided evidence for the impact of HK2 expression levels on cardiovascular diseases, such as MI (Okuyama et al., 2015). Therefore, the role of HK2 in aortic dissection warrants further investigation.

Cadherin-8 (encoded by *Cdh8*) is an integral membrane protein crucial for calcium-dependent intercellular adhesion and has recently been implicated in autism: (Kerschbamer et al., 2022; Nita et al., 2021; Tu et al., 2021; Hurley et al., 2021). Tryptophan hydroxylase 1 (encoded by *Tph1*) is a member of the aromatic amino acid hydroxylase family. Extensive research has indicated that mutations in this gene are associated with an increased risk of various diseases and conditions, including schizophrenia, somatic anxiety, anger-related traits, bipolar disorder, suicidal behavior, and addiction (Karanović et al., 2016; Nielsen et al., 2020; Wigner et al., 2018). However, its connection with cardiovascular disease remains to be fully elucidated.

Gaining insights into the effects of these key genes on inflammatory and immune response, as well as epigenetic regulation, will enable a more comprehensive analysis of the intricate interactions among various molecules. This, in turn, could provide valuable insights for the development of small molecule therapies targeting the corresponding pathways. The establishment of an effective animal model not only helps us to understand the interaction between molecules, but also can be used for experimental verification. Unlike traditional animal models such as rats, mice, and rabbits, a multicenter experiment demonstrated that zebrafish is likely to play a key role in the clinical identification of the pathogenicity of variants of unknown significance (VUS) in ATAAD (Prendergast et al., 2025).

Nevertheless, our research has several limitations. Firstly, the development and validation of our model in this study are based on a retrospective dataset obtained from GEO, which comprises a relatively small number of clinical samples. This limitation may lead to curve overfitting and potentially skew the research results. Secondly, some datasets lack relevant clinical information, such as patient symptoms, complications, hypertension status, genetic factors, and other risk factors. Lastly, the functions of the four hub genes, along with their upstream and downstream pathways in ATAAD, require further verification, which will be the focus of our future work.

## 5 Conclusion

Through the application of bioinformatics and machine learning methodologies, four characteristic genes - *Cdh8*, *Ccl2*, *Hk2*, and *Tph1* - have been preliminarily identified as having a close association with ATAAD. A predictive chart for the clinical diagnosis of ATAAD has been established. This nomogram can serve as a tool for identifying high-risk patients with ATAAD in a clinical setting.

## Data Availability

The datasets presented in this study can be found in online repositories. The names of the repository/repositories and accession number(s) can be found in the article/Supplementary Material.

## References

[B1] Bian X. Jiang H. Meng Y. Li Y. P. Fang J. Lu Z. (2022). Regulation of gene expression by glycolytic and gluconeogenic enzymes. Trends Cell Biol. 32 (9), 786–799. 10.1016/j.tcb.2022.02.003 35300892

[B2] Carr M. W. Roth S. J. Luther E. Rose S. S. Springer T. A. (1994). Monocyte chemoattractant protein 1 acts as a T-lymphocyte chemoattractant. Proc. Natl. Acad. Sci. 91 (9), 3652–3656. 10.1073/pnas.91.9.3652 8170963 PMC43639

[B3] Charo I. F. Myers S. J. Herman A. Franci C. Connolly A. J. Coughlin S. R. (1994). Molecular cloning and functional expression of two monocyte chemoattractant protein 1 receptors reveals alternative splicing of the carboxyl-terminal tails. Proc. Natl. Acad. Sci. U. S. A. 91 (7), 2752–2756. 10.1073/pnas.91.7.2752 8146186 PMC43448

[B4] Chen B. Frangogiannis N. G. (2021). Chemokines in myocardial infarction. J. Cardiovasc Transl. Res. 14 (1), 35–52. 10.1007/s12265-020-10006-7 32415537

[B5] Chen L. Zhang Y. H. Lu G. Huang T. Cai Y. D. (2017). Analysis of cancer-related lncRNAs using gene ontology and KEGG pathways. Artif. Intell. Med. 76, 27–36. 10.1016/j.artmed.2017.02.001 28363286

[B6] Chen Y. Feng Y. Yan F. Zhao Y. Zhao H. Guo Y. (2022). A novel immune-related gene signature to identify the tumor microenvironment and prognose disease among patients with oral squamous cell carcinoma patients using ssGSEA: a bioinformatics and biological validation study. Front. Immunol. 13, 922195. 10.3389/fimmu.2022.922195 35935989 PMC9351622

[B7] Daily P. Trueblood H. W. Stinson E. B. Wuerflein R. D. Shumway N. E. (1970). Management of acute aortic dissections. Ann. Thorac. Surg. 10, 237–247. 10.1016/s0003-4975(10)65594-4 5458238

[B8] Erbel R. Aboyans V. Boileau C. Bossone E. Di Bartolomeo R. Eggebrecht H. (2014). 2014 ESC Guidelines on the diagnosis and treatment of aortic diseases. Kardiol. Pol. 72 (12), 1169–1252. 10.5603/KP.2014.0225 25524604

[B9] Eremia I.-A. Popa M.-I.-G. Anghel C.-A. Stroe T.-A. Eremia E.-A. Marinescu A. N. (2025). Outcomes of surgical versus conservative management in Stanford type a aortic dissection: a single-center retrospective study. Life 15 (3), 462. 10.3390/life15030462 40141805 PMC11943683

[B10] Gawinecka J. Schönrath F. von Eckardstein A. (2017). Acute aortic dissection: pathogenesis, risk factors and diagnosis. Swiss Med. Wkly. 147, w14489. 10.4414/smw.2017.14489 28871571

[B11] Gschwandtner M. Derler R. Midwood K. S. (2019). More than just attractive: how CCL2 influences myeloid cell behavior beyond chemotaxis. Front. Immunol. 10, 2759. 10.3389/fimmu.2019.02759 31921102 PMC6923224

[B12] Guo D. Meng Y. Jiang X. Lu Z. (2023). Hexokinases in cancer and other pathologies. Cell Insight 2 (1), 100077. 10.1016/j.cellin.2023.100077 37192912 PMC10120283

[B13] Hagan P. G. Nienaber C. A. Isselbacher E. M. Bruckman D. Karavite D. J. Russman P. L. (2000). The international registry of acute aortic dissection (IRAD): new insights into an old disease. JAMA 283 (7), 897–903. 10.1001/jama.283.7.897 10685714

[B14] Harris K. M. Nienaber C. A. Peterson M. D. Woznicki E. M. Braverman A. C. Trimarchi S. (2022). Early mortality in type A acute aortic dissection: insights from the international registry of acute aortic dissection. JAMA Cardiol. 7, 1009–1015. 10.1001/jamacardio.2022.2718 36001309 PMC9403853

[B15] He H. Xiao L. Wang J. Guo D. Lu Z. (2023). Aerobic glycolysis promotes tumor immune evasion and tumor cell stemness through the noncanonical function of hexokinase 2. Cancer Commun. Lond. Engl. 43 (3), 387–390. 10.1002/cac2.12404 PMC10009661 36604859

[B16] HeW.YuS.LiJ.ChenZ.ZhangJ.LiuY. (2025). From inflammation to remodelling: a novel BASP1+ monocyte subset as a catalyst for acute aortic dissection. J. Adv. Res. 10.1016/j.jare.2025.03.003 40057028

[B17] Huang X. Zhang G. Feng Y. Zhao X. Li Y. Liu F. (2024). Developing and verifying an effective diagnostic model linked to immune infiltration in Stanford type A aortic dissection. Front. Biosci. Landmark Ed. 29 (9), 318. 10.31083/j.fbl2909318 39344316

[B18] Hurley S. Mohan C. Suetterlin P. Ellingford R. Riegman K. L. H. Ellegood J. (2021). Distinct, dosage-sensitive requirements for the autism-associated factor CHD8 during cortical development. Mol. Autism 12 (1), 16. 10.1186/s13229-020-00409-3 33627187 PMC7905672

[B19] Ince H. Nienaber C. A. (2007). Diagnosis and management of patients with aortic dissection. Heart 93 (2), 266–270. 10.1136/hrt.2005.078550 17228080 PMC1861401

[B20] Karanović J. Ivković M. Jovanović V. M. Pantović M. Pavlović-Janković N. Damjanović A. (2016). Tryptophan hydroxylase 1 variant rs1800532 is associated with suicide attempt in Serbian psychiatric patients but does not moderate the effect of recent stressful life events. Suicide Life Threat Behav. 46 (6), 664–668. 10.1111/sltb.12246 27037949

[B21] Kerschbamer E. Arnoldi M. Tripathi T. Pellegrini M. Maturi S. Erdin S. (2022). CHD8 suppression impacts on histone H3 lysine 36 trimethylation and alters RNA alternative splicing. Nucleic Acids Res. 50 (22), 12809–12828. 10.1093/nar/gkac1134 36537238 PMC9825192

[B22] Kimura N. Futamura K. Arakawa M. Okada N. Emrich F. Okamura H. (2017). Gene expression profiling of acute type A aortic dissection combined with *in vitro* assessment. Eur. J. Cardiothorac. Surg. 52 (4), 810–817. 10.1093/ejcts/ezx095 28402522

[B23] Li Z. Wang J. Yu Q. Shen R. Qin K. Zhang Y. (2022). Identification of immune-related gene signature in Stanford type A aortic dissection. Front. Genet. 13, 911750. 10.3389/fgene.2022.911750 35795203 PMC9252449

[B24] Liu L. Zhao Q. Cheng C. Yi J. Sun H. Wang Q. (2021). Analysis of bulk RNA sequencing data reveals novel transcription factors associated with immune infiltration among multiple cancers. Front. Immunol. 12, 644350. 10.3389/fimmu.2021.644350 34489925 PMC8417605

[B25] Liu Q. Zheng J. Sun W. Huo Y. Zhang L. Hao P. (2018). A proximity-tagging system to identify membrane protein-protein interactions. Nat. Methods 15, 715–722. 10.1038/s41592-018-0100-5 30104635

[B26] Livak K. J. Schmittgen T. D. (2001). Analysis of relative gene expression data using real-time quantitative PCR and the 2(-Delta Delta C(T)) Method. Methods 25 (4), 402–408. 10.1006/meth.2001.1262 11846609

[B27] Matsushima K. Larsen C. G. DuBois G. C. Oppenheim J. J. (1989). Purification and characterization of a novel monocyte chemotactic and activating factor produced by a human myelomonocytic cell line. J. Exp. Med. 169 (4), 1485–1490. 10.1084/jem.169.4.1485 2926331 PMC2189236

[B28] Matthews C. R. Madison M. Timsina L. R. Namburi N. Faiza Z. Lee L. S. (2021). Impact of time between diagnosis to treatment in acute type A aortic dissection. Sci. Rep. 11, 3519. 10.1038/s41598-021-83180-6 33568755 PMC7876041

[B29] Nguyen T. B. Do D. N. Nguyen-Thi M. L. Hoang-The H. Tran T. T. Nguyen-Thanh T. (2022). Identification of potential crucial genes and key pathways shared in Inflammatory Bowel Disease and cervical cancer by machine learning and integrated bioinformatics. Comput. Biol. Med. 149, 105996. 10.1016/j.compbiomed.2022.105996 36049413

[B30] Nielsen D. A. Deng H. Patriquin M. A. Harding M. J. Oldham J. Salas R. (2020). Association of TPH1 and serotonin transporter genotypes with treatment response for suicidal ideation: a preliminary study. Eur. Arch. Psychiatry Clin. Neurosci. 270 (5), 633–642. 10.1007/s00406-019-01009-w 30923939

[B31] Nienaber C. A. Clough R. E. (2015). Management of acute aortic dissection. Lancet 385, 800–811. 10.1016/S0140-6736(14)61005-9 25662791

[B32] Nita A. Muto Y. Katayama Y. Matsumoto A. Nishiyama M. Nakayama K. I. (2021). The autism-related protein CHD8 contributes to the stemness and differentiation of mouse hematopoietic stem cells. Cell Rep. 34 (5), 108688. 10.1016/j.celrep.2021.108688 33535054

[B33] Noble W. S. (2006). What is a support vector machine? Nat. Biotechnol. 24, 1565–1567. 10.1038/nbt1206-1565 17160063

[B34] Okuyama N. Matsuda S. Yamashita A. Moriguchi-Goto S. Sameshima N. Iwakiri T. (2015). Human coronary thrombus formation is associated with degree of plaque disruption and expression of tissue factor and hexokinase II. Circ. J. 79 (11), 2430–2438. 10.1253/circj.CJ-15-0394 26346032

[B35] Pan S. Wu D. Teschendorff A. E. Hong T. Wang L. Qian M. (2014). JAK2-centered interactome hotspot identified by an integrative network algorithm in acute Stanford type A aortic dissection. PLoS One 9 (2), e89406. 10.1371/journal.pone.0089406 24586754 PMC3933461

[B36] Paul A. Mukherjee D. P. Das P. Gangopadhyay A. Chintha A. R. Kundu S. (2018). Improved random forest for classification. IEEE Trans. Image Process 27, 4012–4024. 10.1109/TIP.2018.2834830 29993742

[B37] Prendergast A. Sheppard M. B. Famulski J. K. Nicoli S. Mukherjee S. Sips P. (2025). Modeling thoracic aortic genetic variants in the zebrafish: useful for predicting clinical pathogenicity? Front. Cardiovasc Med. 12, 1480407. 10.3389/fcvm.2025.1480407 40066353 PMC11892108

[B38] Pu Y. Zhou Y. Guo T. Chai X. Yang G. (2025). PANoptosis-related gene biomarkers in aortic dissection. Arch. Biochem. Biophys. 768, 110385. 10.1016/j.abb.2025.110385 40086567

[B39] Ritchie M. E. Phipson B. Wu D. Hu Y. Law C. W. Shi W. (2015). Limma powers differential expression analyses for RNA-sequencing and microarray studies. Nucleic Acids Res. 43, e47. 10.1093/nar/gkv007 25605792 PMC4402510

[B40] Rylski B. Schilling O. Czerny M. (2023). Acute aortic dissection: evidence, uncertainties, and future therapies. Eur. Heart J. 44, 813–821. 10.1093/eurheartj/ehac757 36540036

[B41] Suzuki T. Distante A. Zizza A. Trimarchi S. Villani M. Salerno Uriarte J. A. (2009). Diagnosis of acute aortic dissection by D-dimer: the international registry of acute aortic dissection sub-study on biomarkers (IRAD-Bio) experience. Circulation 119 (20), 2702–2707. 10.1161/CIRCULATIONAHA.108.833004 19433758

[B42] Thind A. S. Monga I. Thakur P. K. Kumari P. Dindhoria K. Krzak M. (2021). Demystifying emerging bulk RNA-Seq applications: the application and utility of bioinformatic methodology. Brief. Bioinform 22, bbab259. 10.1093/bib/bbab259 34329375

[B43] Tu Z. Wang C. Davis A. K. Hu M. Zhao C. Xin M. (2021). The chromatin remodeler CHD8 governs hematopoietic stem/progenitor survival by regulating ATM-mediated P53 protein stability. Blood 138 (3), 221–233. 10.1182/blood.2020009997 34292326 PMC8310427

[B44] Vasquez M. M. Hu C. Roe D. J. Chen Z. Halonen M. Guerra S. (2016). Least absolute shrinkage and selection operator type methods for the identification ofserum biomarkers of overweight and obesity: simulation and application. BMC Med. Res. Methodol. 16, 154. 10.1186/s12874-016-0254-8 27842498 PMC5109787

[B45] Wang W. Chen X. K. Zhou L. Wang F. He Y. J. Lu B. J. (2024). Chemokine CCL2 promotes cardiac regeneration and repair in myocardial infarction mice via activation of the JNK/STAT3 axis. Acta Pharmacol. Sin. 45 (4), 728–737. 10.1038/s41401-024-01447-w 38086898 PMC10943228

[B46] Wigner P. Czarny P. Synowiec E. Bijak M. Białek K. Talarowska M. (2018). Association between single nucleotide polymorphisms of TPH1 and TPH2 genes, and depressive disorders. J. Cell Mol. Med. 22 (3), 1778–1791. 10.1111/jcmm.13459 29314569 PMC5824396

[B47] Xu S. Catapang A. Doh H. M. Bayley N. A. Lee J. T. Braas D. (2018). Hexokinase 2 is targetable for HK1 negative, HK2 positive tumors from a wide variety of tissues of origin. J. Nucl. Med. 60, 212–217. 10.2967/jnumed.118.212365 29880505 PMC8833855

[B48] Yoshimura T. Robinson E. A. Tanaka S. Appella E. Leonard E. J. (1989). Purification and amino acid analysis of two human monocyte chemoattractants produced by phytohemagglutinin-stimulated human blood mononuclear leukocytes. J. Immunol. 142 (6), 1956–1962. 10.4049/jimmunol.142.6.1956 2921521

[B49] Zhang B. Zeng K. Guan R. C. Jiang H. Q. Qiang Y. J. Zhang Q. (2023). Single-cell RNA-seq analysis reveals macrophages are involved in the pathogenesis of human sporadic acute type A aortic dissection. Biomolecules 13 (2), 399. 10.3390/biom13020399 36830768 PMC9952989

[B50] Zhang T. T. Li Q. G. Li Z. P. Chen W. Liu C. Tian H. (2024). Development and validation of a 6-gene signature derived from RNA modification-associated genes for the diagnosis of Acute Stanford Type A Aortic Dissection. J. Geriatr. Cardiol. 21 (9), 884–898. 10.26599/1671-5411.2024.09.007 39483269 PMC11522717

[B51] Zhao Z. Chen T. Liu Q. Hu J. Ling T. Tong Y. (2025). Development and validation of a diagnostic model for Stanford type B aortic dissection based on proteomic profiling. J. Inflamm. Res. 18, 533–547. 10.2147/JIR.S494191 39816951 PMC11734266

[B52] Zhou X. Chen Z. Zhou J. Liu Y. Fan R. Sun T. (2021). Transcriptome and N6-methyladenosine RNA methylome analyses in aortic dissection and normal human aorta. Front. Cardiovasc Med. 8, 627380. 10.3389/fcvm.2021.627380 34124185 PMC8193080

[B53] Zhou Z. Liu Y. Zhu X. Tang X. Wang Y. Wang J. (2020). Exaggerated autophagy in Stanford type A aortic dissection: a transcriptome pilot analysis of human ascending aortic tissues. Genes (Basel) 11 (10), 1187. 10.3390/genes11101187 33066131 PMC7650806

[B54] Zhu Y. Lingala B. Baiocchi M. Tao J. J. Toro Arana V. Khoo J. W. (2020). Type A aortic dissection-experience over 5 decades: JACC historical breakthroughs in perspective. J. Am. Coll. Cardiol. 76 (14), 1703–1713. 10.1016/j.jacc.2020.07.061 33004136

